# We know more and less about love: a comment on Babková and Repiská (2025)

**DOI:** 10.3389/fnbeh.2025.1602582

**Published:** 2025-07-31

**Authors:** Adam Bode, Marta Kowal

**Affiliations:** ^1^School of Archaeology and Anthropology, The Australian National University, Canberra, ACT, Australia; ^2^IDN Being Human Lab, Institute of Psychology, University of Wroclaw, Wroclaw, Poland

**Keywords:** biology, love, romantic love, molecular science, review, comment

## Introduction

Love is an important aspect of human life. It is the glue that keeps families together, ensures the raising of viable offspring, and is the basis for romantic relationship formation (Kowal et al., 2024) and maintenance (Fletcher et al., 2015). It is understudied and the contributions that researchers make to this field should contribute to an accurate understanding of this important phenomenon. There have been circumstances where theories about love (e.g., Fisher, 1998) have been widely adopted despite lack of empirical evidence. This diminishes the work of love researchers and misdirects scientific efforts and inquiry. It also burdens the science of love with the perception that it lacks rigor and evidence. Therefore, it is important that reviews of the biology of love are complete and accurate.

One recent paper by Babková and Repiská (2025) in International Journal of Molecular Sciences aimed to review the molecular underpinnings of love. The authors reviewed primarily endocrinological studies, specifically those related to neurotrophins, cortisol, serotonin, dopamine, endorphins, testosterone, oxytocin, and vasopressin. It is unclear whether the authors are taking an inductive or deductive approach to the topic, for reasons detailed below. It appears to be somewhat inductive in that it relies exclusively on findings related to romantic love. However, it also appears somewhat deductive as it makes overarching claims which are not supported by evidence in text. Additionally, Babková and Repiská (2025) tackled potential “side effects of the modern world” on love experiences. Although we appreciate scholars' interest in love, we would like to comment on several shortcomings of the target paper which we think are important to note and clarify. We focus here on some of the main shortcomings with particular reference to romantic love, one of our areas of expertise.

Our main criticisms of Babková and Repiská (2025) are that the review (i) lacks clarity about what love is and the different types of love, (ii) is incomplete and does not include some of the biopsychological science of love, (iii) makes claims that are not supported by the evidence, and (iv) inadequately supports their claims. We attempted to have this opinion published as a comment in the journal in which the article was published, International Journal of Molecular Sciences. It was desk rejected.

## Lack of clarity about what is meant by “love”

The authors do not provide a definition of love and do not clearly delineate the different types of love. This is important because different types of love (e.g., romantic love, companionate love, maternal love, filial love, love of pets, love of a god, etc.) all have both similar and unique functions (Machin, 2022). For example, romantic love serves a number of reproductive functions (Bode and Kushnick, 2021), whereas love of a pet does not. Additionally, each type of love would be characterized by shared and unique mechanisms that underly their expression (e.g., Rinne et al., 2024). The title of the article suggests that the topic of the review is love, generally, but then the authors rely entirely on studies related to romantic love.

## Incompleteness

The article cites, primarily, research on romantic love. However, there are a number of important studies that are omitted. The authors fail to cite important studies on love that demonstrated activity of dopamine, endorphins, oxytocin, and vasopressin. Connected to the above point on the lack of clarity on the definition of love and which type of love is within the scope of focus, the target article also fails to reference studies on the role of neurochemicals in other types of love (e.g., maternal love).

### Dopamine

The authors do not cite any studies for the claim that dopamine is involved in romantic love. They do cite one hypothesis article (Fisher et al., 2002) that proposed that dopamine plays a role in discriminating among potential partners and focuses courtship activities. There are numerous fMRI studies demonstrating involvement of dopamine rich structures, such as the mesolimbic pathway, in romantic love (for a review see Xu et al., 2015). There has also been one study (Marazziti et al., 2017) demonstrating lower density of lymphocyte dopamine transporter in individuals experiencing romantic love compared to controls, indicating an up-regulated dopamine system in romantic love.

### Endorphins

The authors do not cite any studies to support the claim that opioids are involved in romantic love. Importantly, one study (Ulmer-Yaniv et al., 2016) demonstrated higher levels of beta-endorphins in people in the early stages of a romantic relationship (presumably experiencing romantic love) compared to single controls. The same study also demonstrated higher beta-endorphin levels in new parents compared to singles, suggesting a role for these neuropeptides in parental love as well. Additionally, the authors do not reference the Brain Opioid Theory of Social Attachment (see Machin and Dunbar, 2011). This theory proposes that social bonding, including both romantic and parental attachment, is mediated by the brain's opioid system, providing compelling evidence of endorphins' involvement in love.

### Oxytocin and vasopressin

The authors fail to cite any of the three human studies (Schneiderman et al., 2014, 2012; Ulmer-Yaniv et al., 2016) which are suggestive of increased oxytocin activity in romantic love, one of which (Ulmer-Yaniv et al., 2016) indicates a role of oxytocin in early parental love. The authors also fail to cite studies which provide indirect evidence of vasopressin's involvement in romantic love. Specifically, studies employing fMRI techniques (see Ortigue et al., 2010 for a meta-analysis) implicate vasopressin-rich structures in romantic love and one study employing genetic and fMRI methods (Acevedo et al., 2020) implicates polymorphisms regulating oxytocin and vasopressin receptors in romantic love.

### Maternal love

The second most studied type of love from a biopsychological perspective is maternal love (see Rigo et al., 2019). The authors fail to cite any studies on maternal love, which is a major shortcoming in an article purporting to review the molecular basis of love. Key studies include a meta-analysis of fMRI studies of maternal love (Rigo et al., 2019) and individual studies investigating circulating peptide levels in new parents (Ulmer-Yaniv et al., 2016), and, specifically, new mothers (Feldman et al., 2010).

## Claims unsupported by evidence

The target article proposes a phases model of romantic love running from sexual desire to intimacy and emotional closeness to bonding and commitment. This is not informed by evidence and the authors provide no citation to justify this model. As active researchers of romantic love, we are not aware of any high-quality literature that provides empirical support for this model. We have never seen this model proposed elsewhere.

The authors' claim that the serotonin system is involved in the obsessive aspects of romantic love was drawn from a 2005 review (Meloy and Fisher, 2005) which considered only two fMRI studies of romantic love, relied on an outdated model of mammalian reproduction (Fisher, 1998), did not incorporate one of the serotonin studies of romantic love (Langeslag et al., 2012), and had an inaccurate outdated interpretations of another study (Marazziti et al., 1999). A review (Bode and Kushnick, 2021), hypothesis (Bode, 2023), and empirical study published since the review in question was submitted (Bode et al., 2025) have provided contradictory evidence for the hypothesis that the same serotonergic mechanisms involved in obsessive compulsive disorder are responsible for the obsessive thinking characteristic of romantic love.

## Inadequate support of claims

Key among our concerns is the use of inappropriate citations to justify claims. For example, the target article reads, “Recently, there has also been growing interest in understanding the biological underpinnings of love. A growing body of research on functional magnetic resonance imaging (fMRI), positron emission tomography (PET), and single photon emission computed photography (SPECT) concluded that there is a specialized network of the brain and certain biomarkers involved in love [4, 5, 6].” None of the citations for this claim are reviews of the neuroimaging of love or even studies investigating love. One (Höfer et al., 2013) is a review about testosterone activity in the brain, one (Yen et al., 2023) is a review of neuroimaging techniques, and one (Ko et al., 2013) is a review of brain stimulation and functional imaging. None of these articles include the word “love” in text. To our knowledge, there has only ever been one PET study on romantic love (Takahashi et al., 2015) and no SPECT studies have ever been undertaken. This type of mis-referencing and questionable support for claims is common throughout the article. Table 1 presents some of our concerns about the citations used in the target article.

**Table 1:** Concerns about citations in text of Babková and Repiská (2025).

**Sentence**	**Comment**
“A growing body of research on functional magnetic resonance imaging (fMRI), positron emission tomography (PET), and single photon emission computed photography (SPECT) concluded that there is a specialized network of the brain and certain biomarkers involved in love [4–6].”	None of the citations for this claim are reviews of the neuroimaging of love or even studies investigating love. One (Höfer et al., 2013) is a review about testosterone activity in the brain, one (Yen et al., 2023) is a review of neuroimaging techniques, and one (Ko et al., 2013) is a review of brain stimulation and functional imaging. To our knowledge, there has only ever been one PET study on romantic love (Takahashi et al., 2015) and no SPECT studies have ever been undertaken.
“However, in many cases, definite proof is still lacking and the few human studies on love are limited by selection bias on the duration of a love affair, gender, and cultural differences [8, 9].”	This sentence should be marked as a quotation, since the referred paper by de Boer et al. (2012) wrote: “The limited number of available imaging studies on love and affection is hampered by selection bias on gender, duration of a love affair, and cultural differences.”
“Passion (…) is a synonym for sexual attraction.”	The Authors claim that “Passion (…) is a synonym for sexual attraction.” and cite Fletcher et al. (2015) to support it. This is a narrow view on Passion. According to the Triangular Theory of Love, which the Authors also refer to in their paper, passion is not limited only to sexual attraction: “The passion component refers to the drives that lead to romance, physical attraction, sexual consummation, and related phenomena in loving relationships. The passion component thus includes within its purview those sources of motivational and other forms of arousal that lead to the experience of passion in a loving relationship.” (Sternberg, 1986, p. 119).
“Emotionally, it includes feelings of attraction, both romantic and sexual, distress when the relationship is threatened, longing for reciprocation, a desire for deep connection, and physiological arousal.”	The description of the components the Authors provided seems misleading. Specifically, the Authors attributed “physiological arousal” into “emotional” component of love, whereas it should rather be a separate, physiological component.
“Interestingly, NGF levels have been shown to be significantly higher in those subjects who had recently fallen in love compared to subjects who were single or engaged in a long-lasting relationship [16]. A positive association between the intensity of early romantic feelings and serum levels of NGF has been identified [17].”	16 is a reference to an empirical study, which indeed supports the first sentence. However, the second sentence is referenced to a review, which reported the results of the 16 reference. Now, it seems as if there were more studies supporting the claimed conclusions.
“In fact, women showed a significant and negative correlation between BDNF levels and the avoidance scale”	The referenced review article focuses on the epidemiology of Generalized Anxiety Disorder (GAD) and discusses aspects such as its prevalence, onset, course, comorbidity, symptom specificity, sociodemographic correlates, impairments, and treatment seeking behaviors. It does not, however, refer to brain-derived neurotrophic factor.
“Increased BDNF signaling was also shown to ameliorate symptoms of depression [19].”	Crucial information missing that the cited study investigated this in mice.
“the establishment of a romantic relationship also requires a certain level of calm and trust.”	This sentence is not warranted by any reference. In fact, there are many relationships commenced with extremely high passion, which may be born out of “love at first sight” (Sternberg, 1986, p. 124), so called infatuated love, or those born out of fatuous love, which are “kind of love we sometimes associate with Hollywood, or with whirlwind courtships, in which a couple meets on Day X, gets engaged 2 weeks later, and marries the next month” (Sternberg, 1986, p. 124). Not to mention pre-arranged marriages in a classical sense, which start with an arrangement of family members, with future spouses sometimes seeing each other for the first time during wedding ceremony.
“Inconsistent findings can be attributed to methodological differences. Firstly, the data obtained from female participants were found to be substantially influenced by the specific phase of the menstrual cycle”	None of the points made in these sentences seem to support it. Marazziti and Canale (2004)—“The women had regular menstrual cycles and were not taking contraceptive pills.” Sorokowski et al. (2019) all women were “between the second and fourth day of the menstrual cycle”; Loving et al. (2009) tested only “birth control use” and it was not significantly related to the DVs. Weisman et al. (2015) did not measure nor report results for menstrual cycle. Berger et al. (2016) was as study on men. Therefore, such a conclusion is not warranted by the five cited studies.
“It has been suggested that a mild increase in the beta-endorphin level creates a sense of wellbeing and euphoria often presented at early stages of love [52].”	53 reference is a study on rats: “Dopaminergic inhibition of pituitary β-endorphin-like immunoreactivity secretion in the rat.” Endocrinology, 110(2), 657–659.
“One study investigated the effects of naloxone, an opioid antagonist, on female sexual response. The researchers found that administering naloxone in two separate doses of 2 mg each time led to an increase in the intensity of orgasms and the overall pleasure experienced by the women. This suggests that blocking opioid receptors, which are typically activated by endorphins, can paradoxically enhance certain aspects of female sexual experience. However, the same dose of naloxone (2 mg) administered only once had the opposite effect, inhibiting both sexual arousal and the ability to achieve orgasm.”	There is no reference provided, and therefore, it is not known which study the Authors refer to. We carefully cross-checked any mentions of such a study in the two reviews the Authors cite immediately before and after these sentences, and the only study that could fit this description seems to be this one: Gillman and Lichtigfeld (1983). The effects of nitrous oxide and naloxone on orgasm in human females: a preliminary report. Journal of Sex Research 19 49–57. Sadly, we were not able to access the full paper. Nevertheless, we found the following, more detailed description of this study in Pfaus and Gorzalka (1987, p. 5): “Naloxone has also been reported to produce a dual effect on the subjective experience of sexual pleasure in females asked to masturbate to orgasm. Gillman and Lichtigfeld [64] reported that naloxone (0 4–4.0 mg, IV), given to four adult females, either enhanced or diminished sexual pleasure during orgasm m a dose-dependent fashion. Naloxone or distilled water was administered through an intravenous indwelling catheter during the late preorgasmic phase of sexual arousal (characterized by hyperventilation). In every case, subjects reported that the degree of sexual pleasure immediately before and during orgasm was enhanced when low doses of naloxone were administered (0.4–2.0 mg). One subject reported that on two occasions rejections of naloxone (2.0 mg) produced the most intense orgasms she could remember Higher doses of naloxone
	(above 2.0 mg), however, were reported to diminish the degree of sexual pleasure. In two of the four subjects, higher doses produced an immediate inhibition of sexual arousal that lasted for several minutes before masturbation could be resumed. Control rejections of distilled water had no effect on sexual responsiveness” Based on this description, the target paper description of this study does not seem to be accurate.
“Half of the testosterone amount in females is generated by the ovaries; the rest by the cortex of suprarenal glands [58].”	This is a direct 1:1 quote from the referenced study. Not only should it be marked as such, but also, the sources should rather be examined, as the referenced study supported this claim using prior research and did not establish this in their study. The referenced study: “Half of testosterone amount in females is generated by the ovaries, the rest by the cortex of supra-renal glands (Lobotsky et al., 1964; Wu et al., 2010)” (p. 435).
“Consistently, studies have demonstrated that interactions with young women elicit an increase in salivary testosterone levels also in men, regardless of whether the interaction occurred in a laboratory or a natural environment”	This sentence is missing a reference.
“It is also evident that the concentration of testosterone, whether it is at the current level or that experienced during fetal development, plays a role in determining the specific mating strategies and types of romantic behavior individuals employ. Our previous work showed that plasma testosterone levels in young men negatively correlated with a romantic loving style and selfless altruistic love [71]. Moreover, not only does actual plasma matter, but also prenatal androgen priming can play a role”	The Authors cite their empirical study, in which they found evidence for the links between “the specific mating strategies and types of romantic behavior individuals employ” and current levels of testosterone; unsurprisingly (given the recent meta-analysis) they did not find evidence for the link with the 2D:4D, which they claim is a proxy of prenatal testosterone (but see a recent meta-analysis suggesting that 2D:4D may not be a reliable measure of prenatal testosterone; Sorokowski and Kowal, 2023). Therefore, the Authors should not say that this is “evident” that “the concentration of testosterone, (…) experienced during fetal development, plays a role in determining the specific mating strategies and types of romantic behavior individuals employ.”
“The initial phase of romantic love, characterized by infatuation and mutual attraction, typically transitions into a more stable, secure attachment style marked by loyalty and secure partnership [76].”	This sentence is not supported by the cited reference.
“For instance, studies conducted on American population have shown longitudinal declines in testosterone levels independent of chronological aging due to various reasons such as higher body mass index (BMI), decreased physical activity, environmental factors, etc. [120].”	The cited study investigated men 45–79 years old, so this was rather a specific sample, not an “American population.”
“Moreover, it can alter our capacity to experience love, physical intimacy, and genuine human connection”	Such a statement requires a reference.
“Many studies show that one of the most common consequences of social media addiction is the relationship problem.”	This is a direct 1:1 quote from the referenced study: “Many studies show that one of the most common consequences of social media addiction is the relationship problem.” p. 894, and should be marked as such.
“We found a study indicating that individuals from Western cultures tend to favor verbal communication in expressing romantic affection, whereas East Asians are more inclined to use gift-giving as a means of conveying their romantic feelings [126].”	Reference 126 is a review. The described study should be referenced to a source, that is, Beichen and Murshed (2015).

Table 1 of the target article includes two major shortcomings. The authors intend for the table to provide a summary of the love-related molecule, psychological effect, brain region, and receptor/interacting molecule implicated in love. The authors, however, fail to provide evidence that the psychological effects stated are associated with love. Additionally, despite there being more than 20 fMRI studies of romantic love (Bode and Kowal, 2023), the authors only cite three fMRI studies of romantic love (Aron et al., 2005; Scheele et al., 2013; Song et al., 2015) and one early meta-analysis of love (Ortigue et al., 2010). Each brain region in the table should have been accompanied by a list of citations referring to fMRI studies implicating that specific brain region in love. Because of the poor reliability of fMRI studies (see Eklund et al., 2016; Elliott et al., 2020), it is important to have replicated evidence of specific brain region involvement in romantic love (see Bode and Kushnick, 2021 for an example of how this can be done). For example, a meta-analysis of nine fMRI studies of romantic love (Shih et al., 2022) found only the ventral tegmental area could be reliably associated with romantic love. Simply because a neurotransmitter or neurohormone is associated with love does not mean that it is specifically involved in all regions where that factor is active. The citations provided by the authors are insufficient evidence of involvement of all the stated brain regions in love.

## Other concerns

In addition to the shortcomings detailed above, we note a number of other issues with the article, including contradictory statements, misrepresentation of studies, irrelevant considerations, and the detailed consideration of 2D:4D ratios as a proxy for prenatal testosterone exposure (something which is convincingly in doubt; see Sorokowski and Kowal, 2023). The authors also conflate passion with sexual attraction, and mischaracterize the cognitions, emotions, and behaviors of romantic love.

## Suggestion for researchers

In essence, we found the review by Babková and Repiská (2025) to be incomplete and misleading. As a result, we caution researchers against relying on this review to inform their understanding of the biochemistry of love. Instead, researchers should refer to established and rigorous reviews in the literature (e.g., Bode and Kushnick, 2021) individual studies (see Bode and Kowal, 2023 for a list of biological studies of romantic love), and theory (e.g., Bode, 2023) to guide an appreciation of the biochemistry of romantic love. Additionally, other works can provide insights into the biochemistry of maternal love (Rigo et al., 2019).
